# The antimicrobial peptide alpha defensin correlates to type 2 diabetes via the advanced glycation end products pathway

**DOI:** 10.4314/ahs.v22i1.37

**Published:** 2022-03

**Authors:** Mohammed El-Mowafy, Abdelaziz Elgaml, Naglaa Abass, Amany A Mousa, Mohamed N Amin

**Affiliations:** 1 Microbiology & Immunology Department, Faculty of Pharmacy, Mansoura University, Mansoura, 35516, Egypt; 2 Microbiology & Immunology Department, Faculty of Pharmacy, Horus University, New Damietta, 34517, Egypt; 3 Internal Medicine Department, Faculty of Medicine, Mansoura University, Mansoura, 35516, Egypt; 4 Biochemistry Department, Faculty of Pharmacy, Mansoura University, Mansoura, 35516, Egypt

**Keywords:** Alpha defensing, Advanced glycation end products, Hyperglycemia, Inflammation, Innate immunity, Type 2 diabetes

## Abstract

**Background:**

Diabetes is a serious health problem that results in high mortality rates worldwide. α-defensins are antimicrobial peptides of the innate immune system that contribute to inflammation. However, data on serum levels of α-defensin in patients suffering from type 2 diabetes are limited.

**Objectives:**

This study aimed to assess the possible changes in α-defensin serum levels in patients suffering from type 2 diabetes and to investigate its correlation with relevant biomarkers.

**Methodology:**

Analysis of serum α-defensin levels in 47 type 2 diabetics with diabetic neuropathy, 19 type 2 diabetics with no complications and 19 healthy control subjects by enzyme-linked immunosorbent assay was established. Furthermore, measurement of advanced glycation end products (AGEs) and fasting blood glucose (FBG) serum levels was performed, together with the lipid profile analysis.

**Results:**

The serum levels of α-defensin were higher in patients with and without diabetic neuropathy in comparison to control subjects. In addition, there was a significant correlation between α-defensin serum levels and AGEs and FBG serum levels as well as with the body mass index.

**Conclusions:**

α-defensins are significantly elevated in serum of type II diabetics, and correlate with AGEs serum levels indicating a crosstalk that may aggravate inflammation in type 2 diabetes.

## Introduction

Approximately, there were 1.6 million deaths directly brought about by diabetes worldwide in 2016^1^. Type 2 diabetes mellitus is a worldwide health burden with the defining feature of hyperglycemia^2^. Diabetes is a major cause of cardiovascular morbidity and mortality, renal failure, amputation and blindness^3^.

Type 2 diabetes and its chronic complications have been linked chronic inflammation. Several biomarkers have been reported to be associated with the background chronic inflammation resulting in type 2 diabetes. C-reactive protein and interleukin-6 elevations in plasma were shown to predict the development of type 2 diabetes^4^. Obesity as a metabolic syndrome factor also might participates to the inflammation milieu. Intermediate reasonable exercises were found to decrease serum levels of interleukin-6^5^. Also, neuregulin-4 and uric acid/high-density lipoprotein-cholesterol (HDL-C) ratio elevations were depicted to be possible predictors of poor control in type 2 diabetes^6^, ^7^. Moreover, changes in the ratios of different blood cells have been shown to correlate with type 2 diabetes. Neutrophil to lymphocyte and platelet to lymphocyte ratios were shown to be useful in predicting the development and control levels of type 2 diabetes mellitus^8^, ^9^. Besides, another useful marker for predicting the diabetic renal injury is the monocyte to lymphocyte ratio^10^.

A chronic neurological complication of type 2 diabetes mellitus is diabetic neuropathy. Diabetic neuropathy is a leading reason of high mortality, disability and poor life quality in type 2 diabetics. Hyperglycemia and chronic background inflammation are the key players incorporated in the pathogenesis and progression of diabetic neuropathy^11^. The correct incidence percentage of diabetic neuropathy is underestimated because of difficult assessment^12^. Large nerve fiber damage in diabetic neuropathy causes many symptoms such as numbness, sensory loss and muscle weakness. On the other hand, foot ulcer, anesthesia, pain and several autonomic symptoms are brought about by small nerve fiber damage^13^. Experimental diabetes revealed that oxidative stress is an important inducer of tissue injury that occurs in the peripheral nerves^13^. Moreover, another leading pathway incorporated in diabetic neuropathy is the protein kinase C4.

Advanced glycation end products (AGEs) are heterogeneous compounds derived from protein glycation and their precursors. Protein glycation via the Maillard reaction binds reducing sugars and free amino groups of proteins via nucleophilic addition that forms Schiff's base^14^. Elevated AGEs serum level in type 2 diabetes has been well documented^15^. A main downstream pathway of AGEs is activation of receptors of advanced glycation end products (RAGE). RAGE is a multi-ligand immunoglobulin in the super-family of cell-surface molecules^16^. Activation and translocation of proinflammatory kinases and transcription factors, such as nuclear factor kappaB (NFkB) as well as generation of reactive oxygen species are the best reported sequelae of RAGE activation16. Also, AGEs are related to the progression of diabetic complications, notably nephropathy, retinopathy and neuropathy^16^,^17^.

Defensins are small antimicrobial peptides of the innate immune system^17^. Such peptides contribute to the development of inflammation^18^. Defensins are grouped into two major classes: α- and β-defensins. Human α-defensins involve human neutrophil peptide 1–4 and human intestinal defensins produced by Paneth cells. α-defensins can induce chemotaxis and elevate proinflammatory cytokines in addition to their antimicrobial effects^19^,^20^. Also, α-defensins increase attachment of low-density lipoprotein-cholesterol (LDL-C) to endothelial cells in blood vessels, suggesting their contribution to atherosclerosis^21^. α-defensins originate from neurophil granulocytes; constituting 30%–50% of the granule proteins. These peptides are secreted into the extracellular environment due to degranulation, leakage and cell lysis^22^.

We aimed to assess possible changes in serum levels of α-defensin in type 2 diabetic patients. Moreover, we investigated the possible correlation with AGEs, fasting blood glucose (FBG), lipid profile and the oxidative stress marker, malondialdehyde. We also stretched the scope of our research to the changes in these markers that might take place in diabetic neuropathy compared to type 2 diabetics without neuropathy.

## Materials and Methods

### Patients and sample collection

This work was conducted in line with the code of Ethics of the 1964 Declaration of Helsinki and signed informed consent was obtained from each participant. This research was permitted by the Research Ethics Committee, Faculty of Pharmacy, Mansoura University, Egypt (code: 2020-89). The study was conducted with selected outpatients of the Diabetes Clinic at Specialized Internal Medicine Hospital, Mansoura University, Egypt, between August 2019 and November 2019. Exclusion criteria were infection and other disorders or inflammation that directly increases neutrophil activation leading to α-defensin increases, such as autoimmune disorders, malignancy, smoking, pregnancy, lactation, infected diabetic foot, elevated fasting blood glucose (FBG above 450 mg%), diabetic retinopathy, diabetic nephropathy, cardiovascular disease, anemia (hemoglobin <10 mg/dl), liver disease and renal disease.

A thorough history was taken for all patients with an emphasis on sex, age, duration of diabetes, mode of therapy and symptoms of peripheral neuropathy, e.g., paresthesia, dysesthesia, radicular pain, electric shock pain, motor weakness and anhidrosis. Patients also submitted to a general examination including body mass index (BMI kg/m^2^), pulse, blood pressure and waist circumference (cm) (Table 1). We selected 66 type 2 diabetic patients, classified into two groups. The first group was the diabetic neuropathy group that included 47 patients with peripheral nerve dysfunction without foot ulceration. This group composed of 11 males and 37 females, with mean age (51.59 ± 1.15) years. Diagnosis of peripheral neuropathy was based on loss of pressure perception at two or more sites using a monofilament test or loss of vibration perception, >50 Volts using a neurothesiometer. The second group consisted of 19 diabetic patients, five males and 14 females, without peripheral nerve dysfunction and without other diabetic complications. Mean age of the group was 47.84 ± 2.00 years. Finally, 19 healthy subjects, matched for sex and age, were included as a control grup. Blood samples (5 ml) were collected by clean venipuncture. All patients and subjects were fasting for 12 hours. Blood was set for 15 min at room temperature to clot, then, it was centrifuged for 10 minutes at 600xg for serum isolation.

**Table 1:** Clinical features of the studied patients and subjects (mean ± SE)

	Age (years)	BMI (kg/m2)	Waist Circ. (Cm)	Gender	Sys. BP (mm Hg)	Dia. BP (mm Hg)	Diabetes Dur.(y)
**Diabetic** **Neur.**	51.59±1.15	34.25±0.97	115.12±1.99	F (36) M (11)	125.31±2.19	79.25±1.42	9.14±1.00
**Diabetic**	47.84±2.00	33.27±1.61	109.57±2.03	F (14) M (5)	120.52±5.43	77.36±3.13	7.23±1.35
**Control**	43.15±2.09	32.36±1.36	110.57±3.37	F (14) M (5)	120.00±1.2	80.00±1.04	NA

BMI: body mass index; Dia. BP: diastolic blood pressure; Diabetes Dur.: diabetes duration; Diabetic Neur.: diabetic neuropathy; Sys. BP: systolic blood pressure; Waist Circ.: Waist Circumference.

### Fasting blood glucose, malondialdehyde and lipid profile analysis

The clear non-hemolyzed serum was used immediately for measurement of fasting blood glucose (FBG), malondialdehyde (MDA), total cholesterol (Total-C), triglycerides (TAG) and HDL-C. Also, low-density lipoprotein-cholesterol (LDL-C) was calculated. Approximately one ml of serum was stored at −80 °C for further analysis.

### ELISA assay of α-defensin and AGEs

Serum samples were used for measurement of α-defensin (pg/ml) using a commercially available kit (E1649h, EIAab Science Co Ltd., Wuhan, China). Serum levels of AGEs (ng/ml) were also determined using a commercially available kit (E0263h, EIAab Science Co Ltd., Wuhan, China). Microplate reader (ELx800, BioTek Instruments, U.S.A) and BioTek's Gen5™ Data Analysis Software were used for ELISA measurements.

### Statistical analysis

Data are expressed as mean ± standard error. Comparison between groups was established using one-way ANOVA, followed by Tukey posthoc tests (least significant difference) for inter-group comparisons. Pearson correlation was used for correlation analysis. Microsoft Excel (Microsoft ®OfficeExcel®2016) and SPSS version 25 (Chicago, IL, USA) were used for the statistical calculations. Statistical significance was set at P < 0.05.

## Results

### Levels of AGEs, α-Defensin, MDA and FBG

**Figure 1:**
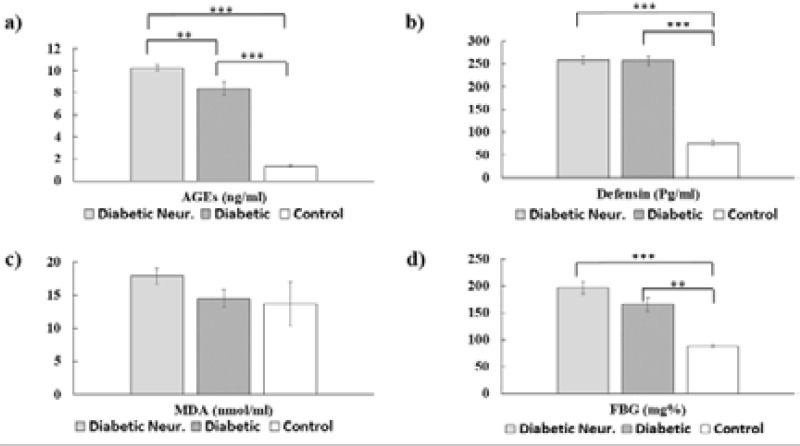
Serum levels of different biomarkers in the study groups. a) AGEs: advanced glycation end products; b) defensin: α-defensin; c) MDA: malondialdehyde; d) FBG: fasting blood glucose; Diabetic Neur.: diabetic neuropathy; (**): P < 0.01; (***): P < 0.001.

AGEs serum levels were significantly elevated in diabetic (P < 0.001) and diabetic neuropathy (P < 0.001) patients in comparison to control subjects (Figure 1a). The increase was dramatic, 7.6 and 6.2-fold, in diabetic neuropathy group and diabetic group with no associated complications compared to control subjects, respectively. Moreover, AGEs levels were significantly elevated in diabetic neuropathy patients in comparison to diabetic patients without neuropathy (P < 0.01). Conversely, α-defensin serum levels significantly increased in diabetic (P < 0.001) and diabetic neuropathy (P < 0.001) patients with 3.4-fold change compared to control subjects (Figure 1b). No significant difference in α-defensin levels between diabetic and diabetic neuropathy patients was observed. Moreover, serum levels of MDA were insignificantly increased in diabetic patients with and without neuropathy in comparison to control subjects (Figure 1c). FBG was significantly higher in patients with neuropathy (P < 0.01) and without neuropathy (P < 0.001) groups in comparison to control subjects (Figure 1d).

### Lipid profile changes

**Figure 2:**
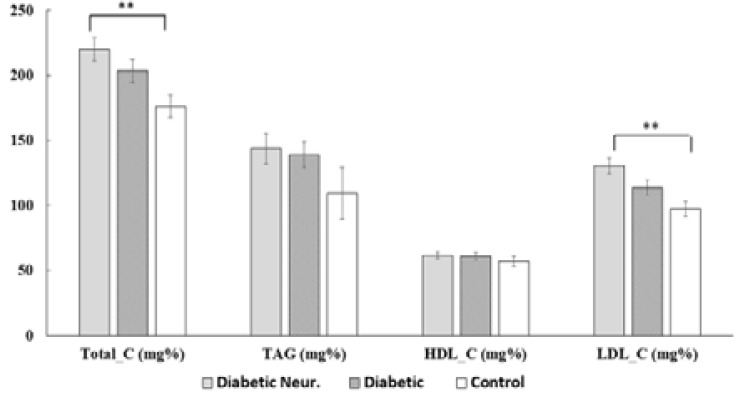
Lipid profile changes in different study groups. Diabetic Neur.: diabetic neuropathy; Total-C: total cholesterol; TAG: triglycerides; LDL-C: low-density lipoprotein-cholesterol, HDL-C: high-density lipoprotein-cholesterol; (**): P < 0.01

Total-C and LDL-C levels significantly increased in diabetic neuropathy (P < 0.01) compared to control subjects (Figure 2). TAG levels were insignificantly elevated in the diabetic patient groups in comparison to control subjects. On the other hand, HDL-C levels were not significant changed among groups.

### α-Defensin significantly correlates with AGEs, FBG, age and diabetic duration

**Figure 3:**
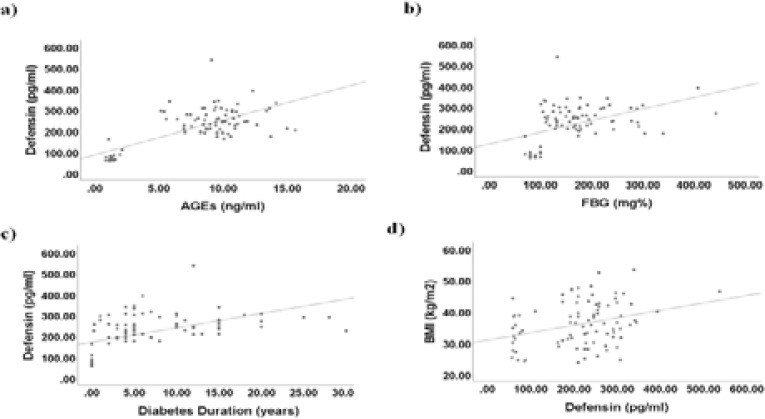
Scatter-dot graphs illustrating significant correlations. a) significant positive correlation between α-defensin (defensin) and advanced glycation end products (AGEs) (Pearson correlation coefficient (r) = 0.703, P < 0.001); b) significant positive correlation between defensin and fasting blood glucose (FBG) (r = 0.441, P < 0.01); c) significant positive correlation between defensin and diabetes duration (r = 0.506, P < 0.01); d) significant positive correlation between α-defensin (defensin) and body mass index (BMI) (r = 0.322, P < 0.01)

Serum levels of α-defensin showed significant correlation with AGEs (r = 0.703, P < 0.001), FBG (r = 0.441, P < 0.01), age (r = 0.566, P < 0.01), diabetes duration (r = 0.506, P < 0.01) and BMI (r = 0.322, P < 0.01) (Figure 3 and Table 2). However, α-defensin did not show significant correlation with either Total-C or MDA.

**Table 2:** Pearson correlation coefficient of α-defensin (defensin) and advanced glycation end products (AGEs) with clinical and biochemical parameters

Parameters	Total_C (mg%)	MDA (nmol/ml)	FBG (mg%)	Age (years)	BMI (kg/m^2^)	Diabetes Dur. (y)
**Defensin** **(pg/ml)**	0.092	-0.205	0.441**	0.566**	0.322**	0.506**
**AGEs (ng/ml)**	0.287**	-0.164	0.528**	0.435**	0.305**	0.457**

Diabetes Dur.: diabetes duration; FBG: fasting blood glucose; MDA: malondialdehyde; Total_C: total_cholesterol

** P < 0.01

### AGEs significantly correlate with Total-C, FBG, BMI, Age and diabetic duration

AGEs levels in serum were significantly correlated with Total-C (r = 0.287, P < 0.01), FBG (r = 0.528, P < 0.01), BMI (r = 0.305, P < 0.01), age (r = 0.435, P < 0.01) and diabetic duration (r = 0.457, P < 0.01). AGEs and MDA in serum were not significantly correlated (Table 2).

## Discussion

We analyzed serum levels of α-defensin in patients suffering from type 2 diabetes with or without diabetic neuropathy. We studied possible correlations between α-defensin and biomarkers of diabetes and metabolic syndrome. Levels of α-defensin were elevated in diabetic patients in comparison to control subjects, and significantly correlated with AGEs, FBG, BMI and diabetes duration. Only Németh et al. analyzed α-defensin serum levels in type 2 diabetes and reported significant elevation compared to controls. However, these authors found that α-defensin serum levels in diabetic neuropathy patients were significantly higher than in diabetic patients with no complications^23^. α-defensin level was higher in diabetic patients with nephropathy than those with cardiovascular complications or neuropathy. Also, there was no significant difference in the cumulative copy numbers of α-defensin gene between patients of type 1 and type 2 diabetes or between controls and diabetics. Moreover, their results showed no positive correlation between the mRNA expression levels and the copy numbers of α-defensin.

Further, a second report presented similar results. However, study subjects were mixed type I and type 2 diabetics^24^. They found no significant changes in defensin concentration in patients in response to low-frequency magnetic field. However, they demonstrated that α-defensin level was increased in healthy subjects in week 5 after exposure to low-frequency magnetic field. A third study showed that thirty percent of type I diabetes patients depict high levels of α-defensin mRNAs in capillary blood, originating from eosinophils^25^. Quantitative RT-PCR performed on FACS-sorted granulocytes identified as CD15dull/CD14weak population confirmed them as the cellular source of α-defensin.

In contrast, no significant difference was observed, in our study, between α-defensin serum levels in diabetic neuropathy group and diabetic group with no associated complications. This result might be attributed to our relatively low number of study subjects.

We show for the first time significant positive correlations between α-defensin and AGEs. AGEs are formed initially by a Maillard reaction between glucose and amino groups of proteins 14. Subsequently, oxidation results in the formation of various compounds collectively termed AGEs. AGEs can be formed via lipid peroxidation^26^, and AGEs levels are elevated in diabetes 27. AGEs bind to specific receptors, receptors of RAGE^28^,^29^. RAGE activation results, prominently, in activation of NFkB and worsening of inflammation in diabetes^30^. RAGE are found in many cell types; most importantly, for our study, are monocytes and macrophages^31^. α-defensins, however, were originally identified in polymorphic neutrophils as part of innate immunity. They are released upon degranulation or lysis and participate in inflammation progression. Also, α-defensins link innate and adaptive immunity, supporting chemotactic activity for T cells, monocytes and dendritic cells^32^. α-defensins are also expressed in monocytes, macrophages and dendritic cells^32^, ^33^, ^34^, ^35^. We speculate that this expression might be the explanation for the correlation between α-defensin and AGEs.

Exaggerated degranulation might lead to inflammation in diabetes. NET-neutrophil extracellular trap-formation is triggered by proinflammatory conditions that occur during hyperglycemia^36^, as well as by α-defensins^37^. Activated neutrophils play a dual role in both expression of adhesion molecules and attachment to the vascular endothelial cells^38^. In concert with this, activated neutrophils also change the composition of their cell membrane and express phosphatidylserine^39^. The activated neutrophils then undergo cell death and produce their cytotoxic components to trigger inflammation. The neutrophil cell death has special features involving the production of web-like DNA including antimicrobial proteins. Strong proinflammatory reactions in the surrounding area are also brought about by neutrophil extracellular traps 40. Hence, neutrophil activation and cell death are involved in the development of diabetic vascular complications^41^.

Upstream signaling molecules in α-defensin and AGEs interplay are not known. Two possibilities exist. First, hyperglycemia may increase AGEs leading to RAGE activation in monocytes and macrophages, elevated NFkB, and eventually elevation of α-defensin in serum. Second, proinflammatory cytokines in diabetes may induce degranulation and release of α-defensins. α-defensins bind to low-density lipoprotein^42^,^43^, thus increasing LDL precipitation in vascular endothelial cells. Finally, lipid peroxidation elevates AGEs levels in serum. We believe that the first possibility is more feasible since α-defensins correlated significantly with FBG in our study, with no significant correlation with any other lipid profile components. We believe that α-defensins correlate mainly with AGEs caused by hyperglycemia but not hyperlipidemia.

Both AGEs and α-defensins correlated significantly with age and diabetes duration. These observations might be explained by a buildup of AGEs, and consequently, α-defensin levels over several years, thus affecting several of these hallmarks^44^. Support for this hypothesis includes an age-dependent increase in browning, fluorescence, cross-linking, insolubility and accumulation of AGEs in lens crystallins^45^.

## Conclusion

α-defensin serum levels are elevated in type 2 diabetics. They might be linked to markers of metabolic syndrome, such as AGEs, FBG and BMI. The results indicate that AGEs-RAGE axis crosstalk aggravate inflammation milieu in type 2 diabetes. More in vitro studies are needed to elucidate the possible signaling stream.

## Data Availability

The data used to support the findings of this study are available from the corresponding author upon request.
